# Italians on the Age of COVID-19: The Self-Reported Depressive Symptoms Through Web-Based Survey

**DOI:** 10.3389/fpsyg.2020.569276

**Published:** 2020-10-16

**Authors:** Michela Balsamo, Leonardo Carlucci

**Affiliations:** Department of Psychological, Health and Territorial Sciences, University of Chieti, Chieti, Italy

**Keywords:** anxiety, coronavirus disease 2019, depression, mental health, worry

## Abstract

The pandemic of coronavirus disease 2019 (COVID-19) has affected the Italian community. The widespread use of quarantine had the desired impact of controlling the epidemic, although it caused many psychological consequences. To date, compliance of the Italian public with voluntary home quarantine has been very high, but little is known about the impact of psychological health on sociodemographic categories during the quarantine. The purpose of this study was to assess the prevalence of depressive symptoms in specific sociodemographic categories during the COVID-19 quarantine lockdown and the potential factors that contribute to, or mitigate, these effects. In the very early stage of the nationwide lockdown, 3,672 quarantined Italian adult residents (65% females, ranging from 18 to 85 years) participated in a web-based cross-sectional survey, including measures of depressive symptoms, which were measured by the Teate depression inventory, and state anxiety levels. The overall prevalence was 27.8% for moderate and 9.3% for severe levels of depressive symptoms. A generalized logistic model was used to identify the factors associated with mental health problems. Among these factors, sociodemographic variables (e.g., sex, age, employment status) and adherence to quarantine guidelines were analyzed. Females, younger people, students, singles, residents in northern Italy, people who were reluctant to adhere to quarantine guidelines, and people less worried about being infected with COVID-19 were at high risk of developing depressive symptoms during the COVID-19 epidemic, also after controlling for state anxiety. These findings showed that public levels of depressive symptoms did not increase the greater likelihood of being infected. Our study suggested that the monitoring of psychological outcomes for outbreaks could identify groups at higher risk of psychological morbidities due to the current pandemic in order to target future psychological interventions for implementation.

## Introduction

The pandemic of coronavirus disease 2019 (COVID-19), caused by the severe acute respiratory syndrome coronavirus 2 (SARS-CoV-2, previously known as 2019-nCoV) has affected the Italian community since late January. According to the Imperial College COVID-19 Response Team (Ferguson et al., 2020), cumulatively, 5.9 (1.9–15.2) million people had been infected as of March 28, giving an infection rate of 9.8% (3.2–25%) of the Italian population.

To contain the rapid spread of this pandemic, the Italian Government ordered nationwide lockdown by March 11: all public places were closed (included educational, religious, and public/cultural institutions, such as schools, universities, museums, and law courts), all public events and any form of congregation were banned, and a distance of at least 1 m had to be maintained (Government of Italy, 2020). All Italian people were in quarantine at home (#iorestoacasa) until May 4: people had to stay at home apart from essential tasks. The slowing growth in daily reported deaths in Italy was consistent with the significant impact of these restrictions. The effective reproduction number, Rt, dropped to close to 1 around the start of the lockdown, with 38,000 (13,000–84,000) deaths averted. The widespread use of quarantine had the desired effect of controlling the epidemic, this was also due to the fact that the compliance of the Italian public with quarantine guidelines had been very high (Carlucci et al., 2020).

Yet, the pandemic created a breeding ground for direct psychological consequences, suddenly throwing many individuals into daily lives filled with health threats, existential depression, and generalized stress (Holmes et al., 2020). A recent review of the psychological impact of quarantine, due to earlier outbreaks, suggested that there were high rates of negative psychological effects among the public, including post-traumatic stress symptoms, persistent depression, substantial anger, panic attacks, and suicidality (Liu et al., 2003; Maunder et al., 2003; Brooks et al., 2020).

Social distancing and isolation exacerbate the burden of stress, and often cause effects on immune, cardiovascular, and mental health because these measures frustrate the deep-seated human instinct to connect with others. On this point, social connection helps people to regulate negative emotions, remain resilient during difficult times, and cope with stress (Rimé, 2009; Hawkley and Cacioppo, 2010; Haslam et al., 2012; Doré et al., 2017; Jetten et al., 2017).

Remarkably, the mental impact of quarantine can depend largely on the characteristics of participants and the quarantine variables selected. As documented by Reynolds et al. (2008), Taylor et al. (2008), and Brooks et al. (2020), while compliance with quarantine guidelines requirements are significant factors behind a higher level of post-traumatic stress disorder (PTSD), healthcare workers were more likely to be affected than the public.

In Italy, in the early phase of quarantine (7–10 days after the decree of nationwide lockdown), deleterious consequences on the population’s psychological health were analyzed in a nationally representative survey of 3,452 participants (Barari et al., 2020). Different demographic groups were struggling with different aspects of quarantine. Older adults expressed worry or anxiety, while those who were likely working parents (40–49 years) cited consistent economic distress and struggles with home-schooling and smart-working, compared to other groups. Younger people were struggling with increased boredom, perceived immobility, and conflicts within family, while vulnerable groups, like the elderly and health-compromised people, cited consistent loneliness relative to others. Overall, the average level of anxiety surrounding the crisis in the Italian population was high: no respondents reported being completely without anxiety.

According to Barari et al. (2020), the negative psychological consequences of the quarantine were beginning to wear on people and seemed likely to become more serious over time.

Further findings derived from an online survey (Mazza C. et al., 2020) showed the prevalence of psychiatric symptoms in 2,766 participants drawn from the general population from March 18 to 22 2020. Female gender, negative affect, and detachment were associated with higher levels of stress, anxiety, and depression. Having an acquaintance infected was associated with increased levels of both depression and stress, whereas a history of stressful situations and medical problems was associated with higher levels of anxiety and depression. Finally, those with a family member infected and young people who had to work outside their domicile presented higher levels of anxiety and stress, respectively.

Thus, it is important that the potential advantages of home quarantine are weighed against the possible mental costs (Rubin and Wessely, 2020; Torales et al., 2020). Quarantine as an efficacious public health measure also needs to lower the psychological strain associated with it.

### Aims of This Study

Research evidence aims of this study were to explore (1) the likely effects of quarantine on mental health (anxiety and depressive symptoms), immediately after the nationwide lockdown issued by the Italian Government, and (2) the factors that contribute to, or mitigate, these consequences.

Among these factors, sociodemographic variables (gender, age, employment status, marital status, education, geographic area, and income per year), worry about being affected by COVID-19, and adherence with quarantine guidelines were analyzed. Depression was the principal outcome, while anxiety was used as a covariate, given its close association with depression (Clark and Watson, 1991; Barlow and Campbell, 2000).

## Materials and Methods

### Sample and Procedure

Respondents were Italian quarantined adults aged 18 and older with access to a networked computer. An online cross-sectional study was conducted from March 21 to 26, immediately after the nationwide lockdown issued by the Italian Government on March 11 (#iorestoacasa). A virtual snowball sample via social media was used within a wider web-based study including other psychological measures (Carlucci et al., 2020).

This study has been approved by the Department of Psychological Sciences, Health and Territory, University of Chieti, Italy Review Board. Written informed consent was obtained from all the participants included in the study.

### Measures

#### Sociodemographic Variables

General information concerning sex, age, education, marital status, geographic area and region, employment status, yearly income, and health status including history of psychiatric illnesses and medical problems (e.g., hospitalizations) were collected.

#### Depression

The 21-item Teate Depression Inventory (TDI; Balsamo et al., 2014, 2018b), developed via Rasch analysis (Rasch, 1960), was employed to evaluate depressive symptoms in participants in the past 2 weeks. Respondent answers were measured on a 5-point Likert scale ranging from “*never*” to “*always.*” Cronbach’s α coefficient in our study was 0.90.

#### Anxiety

The 21-item state scale of the State-Trait Inventory for Cognitive and Somatic Anxiety (STICSA) (Ree et al., 2008; Balsamo et al., 2016; Carlucci et al., 2018) was administered to evaluate cognitive (e.g., “I have trouble remembering things”) and somatic (e.g., “My muscles are tense”) symptoms of state anxiety. Individuals rated how often a statement was true in the past 2 weeks, from 1 “*not at all*” to 4 “*very much so*.” Cronbach’s α coefficient was 0.89.

#### Adherence to Quarantine Guidelines

Adherence to quarantine guidelines in response to the COVID-19 outbreak was measured by a global index composed of 11 items classified into preventive (i.e., handwashing with soapy water/alcohol-based solution) and avoidant (i.e., avoidance of gatherings in public or open to public places, handshaking) disease behaviors (Carlucci et al., 2020). Respondents were asked about the frequency of which they had carried out quarantine restrictions on a 5-point Likert scale from 0 “*never*” to 4 “*always*.” Cronbach’s α coefficient was 0.70.

#### Worry

Worry about being infected with COVID-19 was assessed by a single item drawn from a multidimensional questionnaire of risk perception for the COVID-19 infectious disease outbreak. Responses were classified according to three levels of worry severity: none (“*Not worried at all*”), moderate (“*Slightly worried*”), and quite a lot (“*Really worried*”).

### Data Analysis

Descriptive statistics were computed for sociodemographic characteristics, physical symptoms and health service utilization variables, knowledge and concern-related variables, precautionary measure variables, and additional health information variables. Prevalence of depressive and anxiety symptoms during the COVID-19 outbreak in the Italian population were also computed for sex and age. In line with similar studies (e.g., Giallonardo et al., 2020; Mazza C. et al., 2020; Wang et al., 2020), the TDI outcome score was categorized into “minimal,” “mild,” “moderate,” and “severe” depression levels (Balsamo and Saggino, 2014). A STICSA-S score of 40 points or greater was indicated as the cut-off point for the presence of anxiety symptoms (Van Dam et al., 2013).

Sensitivity analysis was conducted using the Hmisc R package (Harrell and Dupont, 2006) in order to assess the power and sample size of ordinal outcomes under the proportional odds ordinal logistic model.

Next, generalized linear regression (GLMs) was applied to the explanatory model to analyze whether the severity of depression during the COVID-19 quarantine could be predicted by high levels of adherence to quarantine guidelines and worry about being infected with COVID-19, and by sociodemographic variables (Model 1), that resulted as significant in our previous study (Carlucci et al., 2020). Since depression was measured in terms of severity levels, we specified multinomial (ordinal) as the distribution and cumulative logit as the link function.

Predictors were selected according to a two-step process. Firstly, a potential set of considered variables were correlated with the outcome variable. All the potential variables that correlated significantly with the outcome were selected as predictors in the GLMs model. Hence, based on the test of model effects (Wald chi-square statistic and *p*-values), the predictors were compared (Guisan et al., 2002). Only the resulting significant predictors (*p* < 0.05) were retained in the model.

A Wald test (and its 95% confidence interval) based on robust estimates of the coefficients and covariance matrix were used to assess the models, and residual deviance as a goodness-of-fit statistic was applied to evaluate model overdispersion (McCullagh, 2018). The model with the deviance/df ratio closest to the unit was retained as the most parsimonious model (McCullagh, 2018).

In addition, due to the high comorbidity between depression and anxiety symptomatology (Clark and Watson, 1991; Barlow and Campbell, 2000), the model was re-estimated controlling for anxiety as the covariate (Model 2), in order to increase the ability to detect differences on dependent variables (depression severity levels) by an independent variable inserted as the covariate. Differences between the two models were interpreted in terms of unique contribution of any independent variable on depression severity symptoms. The data were statistically analyzed with SPSS for Windows 21.0 (IBM Corp, 2018). Statistical significance was set by *p*-values of less than 0.05.

## Results

### Demographic Characteristics

The characteristics of the participants are shown in Table 1. Of the 3,672 respondents, 1,282 (34.9%) were male and 2,390 (65.1%) were female. The mean (±SD) age of the participants was 33.27 ± 14.29 years. A total of 45.2% of them were located in the south of Italy (*N* = 1660). Among these, 1,817 (49.5%) respondents held a high school diploma, while 1,535 (41.8%) held a higher education qualification (bachelor/master/doctorate). In terms of occupational status and income per year, 1,138 (31.20%) participants were students, 232 (6.3%) were healthcare workers, and 1,742 (47.4%) were employed. Concerning marital status, 2,249 (61.2%) participants were unmarried/single, 901 (24.5%) were married, 103 (2.8%) were divorced/separated, 366 (10%) were cohabiting, and 53 (1.4%) were widowed.

**TABLE 1:** Frequencies and percentages of the demographic characteristics of the study sample (*N* = 3,672).

**Variables**	**n (%)**
**Sex**	
Male	1282 (34.9)
Female	2390 (65.1)
**Age**	
18–29	1995 (54.3)
30–39	723 (19.7)
40–49	404 (11)
50–59	261 (7.1)
Over 60	289 (7.9)
**Marital status**	
Single	2,249 (61.2)
Married	901 (24.5)
Divorced/separated	103 (2.8)
Cohabiting	366 (10)
Widowed	53 (1.4)
Geographic area*	
North-west	897 (24.4)
North-east	338 (9.2)
Central	522 (14.2)
South	1,660 (45.2)
Islands	165 (4.5)
**Employment status**	
Unemployed	416 (11.3)
Retired	144 (3.9)
Student	1,138 (31)
Healthcare professional	232 (6.3)
Employee	1,742 (47.4)
**Adherence to quarantine**	
High	1,787 (48.66)
Low	1,885 (51.34)
**COVID-19-related worry**	
None	278 (7.57)
Moderate	1,068 (29.09)
Quite a lot	2,326 (63.34)

*Missing values n = 90 (2.5%).

Most of the subjects had a high level of health: 2,616 (71.2%) were found to show no physical disease, while 24 (0.7%) were detected as “fragile,” having more than three diseases, and with a long history of chronic medical illness. A total of 967 (26.3%) participants had previously carried out psychotherapeutic treatment. Among these, 97.2% of respondents had carried out at least one psychotherapy treatment (individual, family/couple, and/or group treatment), 20.9% had undergone psycho-pharmacological treatment, and 0.6% had participated in other psychological treatments.

Most (84.2%) of the participants spent their quarantine period with family members. A total of 1,787 (48.7%) were found to be highly adherent to quarantine guidelines, and 2,326 (63.34%) reported that they were worried about being infected with COVID-19.

A sample size of 3,672 was used for the statistical power analyses, and a 1:2 odds ratio was used as a baseline. The alpha level used for this analysis was *p* < 0.05. The *post-hoc* analyses showed that the statistical power for this study was 0.859. Thus, there was an adequate power at the moderate to large effect size level. An N of approximately 4,182 would be needed to obtain statistical power at the recommended 0.90 level (Cohen, 1988).

### Prevalence of Depressive Symptoms, and State Anxiety During the COVID-19 Outbreak Stratified by Sex and Age

The overall prevalence was 6.4% for severe, 24.2% for moderate, 39.7% for mild, and 29.7% for minimal depressive symptoms. The overall prevalence of anxiety symptoms was 13.6%, using the cut-off of >40. Taking together, the prevalence of depressive symptoms and state anxiety severity was significantly higher in female participants, and those younger than 30 years compared to participants aged 31 years or older (*p* < 0.001, as shown in Tables 2, 3). In addition, those who received psychotherapeutic treatment in the past reported higher severity levels of depressive [χ^2^ = 45.58 (3), *p* < 0.001] and anxiety [χ^2^ = 47.43 (1), *p* < 0.001] symptoms relative to the general public.

**TABLE 2:** Prevalence of depressive severity levels, and state anxiety during the COVID-19 outbreak in the Italian population stratified by sex (*N* = 3,672).

		**Total**	**Male**	**Female**	**χ^2^**	***P*-value**
		**n (%)**	**n (%)**	**n (%)**		
TDI severity levels	Minimal	906 (24.7)	381 (29.7)	525 (22.0)	47.79	<0.001
	Mild	1407 (38.3)	509 (39.7)	898 (37.6)		
	Moderate	1019 (27.8)	310 (24.2)	709 (29.7)		
	Severe	340 (9.3)	82 (6.4)	258 (10.8)		
STICSA-S^*a*^	No	2944 (80.2)	1108 (86.4)	1836 (76.8)	48.45	<0.001
	Yes	728 (19.8)	174 (13.6)	554 (23.2)		

*TDI, Teate Depression Inventory; STICSA-S, State-Trait Inventory for Cognitive and Somatic Anxiety—State Scale. ^*a*^STICSA-S was defined as individuals who scored ≥ 40 points (Van Dam et al., 2013).*

**TABLE 3:** Prevalence of depressive severity levels, and state anxiety during the COVID-19 outbreak in the Italian population stratified by age groups (*N* = 3,672).

		**18–29**	**30–39**	**40–49**	**50–59**	**Over 60**	**χ^2^**	***P*-value**
		**n (%)**	**n (%)**	**n (%)**	**n (%)**	**n (%)**		
TDI severity levels	Minimal	309 (15.5)	225 (31.1)	144 (35.6)	107 (41.0)	121 (41.9)		
	Mild	738 (37.0)	280 (38.1)	164 (40.6)	104 (39.8)	121 (41.9)	330.69	<0.001
	Moderate	679 (34.0)	165 (22.8)	85 (21.0)	46 (17.6)	44 (15.2)		
	Severe	269 (13.5)	53 (7.3)	11 (2.7)	4 (1.5)	3 (1)		
STICSA-S^a^	No	1525 (76.4)	595 (82.3)	349 (86.4)	224 (85.8)	251 (86.9)	42.69	<0.001
	Yes	470 (23.6)	128 (17.7)	55 (13.6)	37 (14.2)	38 (13.1)		

*TDI, Teate Depression Inventory; STICSA-S, State-Trait Inventory for Cognitive and Somatic Anxiety—State Scale. ^*a*^STICSA-S was defined as individuals who scored ≥ 40 points (Van Dam et al., 2013).*

### Predictors of Depressive Symptoms During the COVID-19 Outbreak

Preliminarily, nonparametric correlations (*Spearman’s rho*) were performed in order to select independent variables as predictors in the GLMs. As expected, all the sociodemographic variables, as well as worry were found to correlate negatively with the outcome variable (depressive symptom severity levels) ranging from *rho* = 0.298 (age, *p* < 0.01) to *rho* = −0.040 (education, *p* < 0.05), except for sex and worry (*rho* = 0.113, *p* < 0.01 and 0.037, *p* < 0.05, respectively).

Parameter estimates of the generalized linear model and the exponentiated values of the coefficients [the “Exp(B)” column] are displayed in Table 4. The first model resulted in an underestimation of the data with no statistical association for education [Wχ^2^(*df*) = 3.19 (3), *p* = 0.372], and income per year [Wχ^2^(*df*) = 7.306 (3), *p* = 0.063] as predictors of depressive symptoms. To improve model fit, we discharged them and re-estimated the model. The Omnibus test [χ^2^(*df*) = 526.21 (20); *p* < 0.001], and residual deviance/df ratio (deviance/df = 1.038) of the re-estimated models suggested that the refined model fit significantly better than the proposed model (McCullagh, 2018). Female participants showed significantly higher depression scores compared to male participants (β = 0.490; SE = 0.068; *p* < 0.001), with a greater risk of depressive symptoms (odds ratio, 1.632 [95% CI, 1.427–1.866]). Participants aged from 30 years and above reported significantly lower levels of depression scores compared to the younger respondents (β = −0.647/−0.344; SE = 0.186/.101; *p* < 0.001), with a decreased risk of depressive symptoms (odds ratio range: 0.524/0.709 [95% CI, 0.363–0.755/0.581–0.864]). Likewise, widowed, cohabiting, and married participants were less likely to experience depressive symptoms than single/unmarried participants, with a decreased risk of depressive symptoms (odds ratio: 0.521 [95% CI, 0.400–0.679]). Concerning geographic area, participants living in the south of Italy had significantly lower depression scores (β = −0.176; SE = 0.080; *p* < 0.05) compared to residents in the north-west, with a decreased risk of depressive symptoms (odds ratio: 0.839 [95% CI, 0.717–1.981]). Students (β = 0.360; SE = 0.118; *p* < 0.01) and healthcare professionals (β = −0.510; SE = 0.160; *p* < 0.001) were slightly and/or not depressed compared to unemployed participants, respectively. The first group displayed a decreased risk of depressive symptom severity (odds ratio: 0.600 [95% CI, 0.438–0.822]), while the latter showed a higher risk of depression symptoms (odds ratio: 1.358 [95% CI, 1.077–1.713]) compared to the unemployed. Next, those who reported to adhere to the quarantine guidelines had significantly lower levels of depressive symptoms those who were not adherent (β = −0.502; SE = 0.067; *p* < 0.001), with a decreased risk of depressive symptoms (odds ratio: 0.606 [95% CI, 0.531–0.690]). Lastly, participants less worried about being infected with COVID-19 (β = −0.517; SE = 0.127; *p* < 0.001) had significantly lower level of depressive symptoms than those who reported to be quite worried, with a decreased risk of depressive symptoms (odds ratio: 0.596 [95% CI, 0.464–0.765]).

**TABLE 4:** Results of GLMs model 1 (*N* = 3,672).

**Characteristic**	**Depressive symptoms**						
	**β**	**SE**	**Wald χ^2^**	**Sign.**	**Exp(β)**	**95% CI Exp(β)**	
**Sex**							
Female	0.490	0.068	51.343	0.001*	1.632	1.427	1.866
Male					1 (Reference)		
**Age**							
over 60	–0.647	0.186	12.044	0.001*	0.524	0.363	0.755
50–59	–0.607	0.160	14.271	0.001*	0.545	0.398	0.747
40–49	–0.488	0.128	14.384	0.001*	0.614	0.477	0.790
30–39	–0.344	0.101	11.632	0.001*	0.709	0.581	0.864
18–29					1 (Reference)		
**Marital status**							
Widowed	–0.760	0.271	7.815	0.005**	0.468	0.275	0.797
Cohabiting	–0.303	0.112	7.302	0.007**	0.739	0.593	0.920
Divorced/separated	–0.318	0.217	2.138	0.144	0.728	0.475	1.114
Married	–0.426	0.110	14.906	0.001*	0.653	0.526	0.811
Single					1 (Reference)		
**Geographic area**							
Islands	0.134	0.156	0.730	0.393	1.143	0.841	1.554
South	–0.176	0.080	4.823	0.028**	0.839	0.717	0.981
Central	–0.187	0.105	3.179	0.075	0.829	0.675	1.019
North-east	0.117	0.118	0.968	0.325	1.124	0.890	1.419
North-west					1 (Reference)		
**Occupational status**							
Employee	–0.141	0.104	1.823	0.177	0.868	0.707	1.066
Healthcare professional	–0.510	0.160	10.102	0.001*	0.600	0.438	0.822
Student	0.306	0.118	6.691	0.010**	1.358	1.077	1.713
Retired	0.014	0.223	0.004	0.949	1.015	0.655	1.572
Unemployed					1 (Reference)		
**Adherence to quarantine**							
High	–0.502	0.067	56.098	0.001*	0.606	0.531	0.690
Low					1 (Reference)		
**COVID-19-related worry**							
None	–0.517	0.127	16.463	0.001*	0.596	0.464	0.765
Moderate	–0.251	0.072	12.17	0.001*	0.778	0.675	0.896
Quite a lot					1 (Reference)		

*p < 0.001; **p < 0.05. The Exp(β) or Odds ratio and β values (95%Wald CI) were derived from generalized linear regression (logistic ordinal) COVID-19, coronavirus disease 2019.

As expected, a preliminary analysis showed that depression and anxiety symptoms shared approximately 38% of the common variance, as derived by the Spearman rho coefficient (rho = 0.62, *p* < 0.001). Thus, state anxiety, as measured by the state STICSA, as the covariate was inserted in our model (Model 2).

Compared to the previous model, no statistical differences were found in sex, age, and adherence level to quarantine guidelines groups when predicting depression symptom severity, when controlling for anxiety (see Appendix A). Statistically significant differences were maintained in depression symptom severity for marital status, geographic area, and occupational status groups, after controlling for anxiety.

In detail, divorced/separated participants were less likely to experience depressive symptoms compared to single people, with a significant decreased risk of depressive symptoms (odds ratio: 0.654 [95% CI, 0.434–0.983]). On the other hand, no statistical differences on depressive symptom severity was found in cohabiting participants compared to unmarried participants in the second model.

Concerning geographic area, the participants living in central Italy were found less likely to experience severe depressive symptoms (odds ratio: 0.794 [95% CI, 0.641–0.984]) compared to those living in north-west Italy, after removing anxiety effects. Far from the previous model, the healthcare professionals’ group were not found to differ from other occupational groups in predicting high levels of depressive symptoms compared to unemployed participants. Interestingly, participants “moderately” worried about being infected with COVID-19 were more prone to experience high levels of depressive symptoms (odds ratio: 1.252 [95% CI, 0.953–1.643]) compared to participants who were “quite a lot” worried, after controlling for the state anxiety effect.

### Predictors of Depressive Symptoms During the COVID-19 Outbreak in Subsample of Participants With Psychotherapeutic Treatment History

We fitted both the GLMs models (without and with anxiety as the covariate) in the subsample of participants with a psychotherapeutic treatment history. No substantial differences were found in both the model’s goodness of fit (deviance/df = 1.048 vs. 0.911). However, differences in the estimates and standard errors of the two models were found for the model with anxiety as the covariate vs. the model without the covariate (see Appendix B).

In line with models tested on the entire sample, in Model 1, female participants showed significantly higher depressive symptoms scores compared to male participants (β = 0.310; SE = 0.151; *p* < 0.05), with a greater risk of depressive symptoms (odds ratio, 1.364 [95% CI, 1.014–1.834]). Participants aged from 30 to 39 and 50 to 59 years reported significantly lower levels of depressive symptoms scores compared to the younger participants (β = −0.543/−0.696; SE = 0.187/0.339; *p* < 0.05), with a decreased risk of depressive symptoms (odds ratio range: 0.581/0.449 [95% CI, 0.403–0.839/0.256–0.971]).

Likewise, cohabiting (β = −0.526; SE = 0.193; *p* < 0.01) and married (β = −0.446; SE = 0.195; *p* < 0.05) participants were less likely to have experienced depressive symptoms compared to single participants, with a decreased risk of depressive symptoms (odds ratio: 0.591/0.640 [95% CI, 0.404–0.863/0.433–0.946]). Healthcare professionals (β = −0.797; SE = 0.280; *p* < 0.01) were less depressed compared to unemployed participants, with a decreased risk of depressive symptom severity (odds ratio: 0.450 [95% CI, 0.260–0.780]). Next, those who reported to adhere to the quarantine guidelines had significantly lower levels of depressive symptoms compared to those who were not adherent (β = −0.475; SE = 0.133; *p* < 0.001), with a decreased risk of depressive symptoms (odds ratio: 0.622 [95% CI, 0.479–0.809]).

No statistical association was found for worry about being infected with COVID-19 and geographic area.

Surprisingly, in Model 2, no statistically significant differences were maintained in depression symptoms for the sex, marital status, and occupational status groups, after controlling for anxiety. Participants aged from 30 to 39 years reported significantly lower levels of depression scores compared to the younger participants (β = −0.5.04; SE = 0.194; *p* < 0.01), with a decreased risk of depressive symptoms (odds ratio range: 0.604 [95% CI, 0.413–0.884]). Concerning geographic area, the participants living in south Italy were less likely to experience severe depressive symptoms (odds ratio: 0.688 [95% CI, 0.493–0.962]) compared to those living in north-west Italy, after removing anxiety effects. As expected, participants “moderately” and “none” worried about being infected with COVID-19 were more prone to experience high levels of depressive symptoms (odds ratio: 1.757/1.722 [95% CI, 1.292–2.391/1.063–2.791]) compared to participants who were “quite a lot” worried, after controlling for the state anxiety effect.

## Discussion

Quarantine has been used extensively in all countries of the world to lower the spread of the COVID-19 infection and to protect individuals’ health, at different times (Sohrabi et al., 2020).

Quarantine includes the separation and restriction of movement of people who have potentially been exposed to a contagious disease to ascertain if they become unwell, so reducing the risk of them infecting others. It is an unpleasant experience for those affected (Hiremath et al., 2020). Imposed isolation and separation from loved ones, loss of mental health needs (freedom, social contacts, stimulation), uncertainty over disease status, family conflict, and boredom can, on occasion, contribute to the onset of psychological disorders (Brooks et al., 2020). Due to the fact that the psychological impact of quarantine depends largely on the characteristics of participants and the quarantine variables selected, several sociodemographic characteristics have been selected here, with depressive symptoms measured by the TDI as the outcome. In the second model, anxiety, as measured by the STICSA state scale, was inserted as the covariate, given the close relationship with depression (Brooks et al., 2020).

About gender, depressive symptoms were more likely to occur in female participants, with a risk of developing depressive symptoms higher 1.6 compared to male participants in our sample. This finding was in accordance with studies by Qiu et al. (2020) and Wang et al. (2020) among the Chinese general population in the first 2 weeks following the outbreak, as well as Broche-Pérez et al. (2020) among the Cuban population. Also, among the Italian general population higher levels of psychological distress were reported in the female gender compared to their male counterparts (Ho et al., 2020; Mazza C. et al., 2020; Rossi et al., 2020).

Sex differences in depression were not caused by a higher prevalence of COVID-19 infection in women because mortality and vulnerability to the COVID-19 disease indicated that more men are dying from COVID-19 (Lancet, 2020). Thus, these differences seem to be caused by the fact that women carry a different kind of burden from this epidemiological emergency. Gender inequities disproportionately affect the well-being and economic resilience during lockdown. Households are under strain, but children and elderly care, as well as housework generally fall on women (Cluver et al., 2020). By increasing caregiving needs, COVID-19 has intensified the pressure on women to uphold prescriptive feminine norms. Women have to bear more of the burdens of providing additional support for children’s distance learning, and alleviating children’s emotional tedium, isolation, and anxiety of shelter-in-place (Rosenfeld et al., 2020).

In addition, increased intimate partner violence has grown during the quarantine due to COVID-19 because women are required to stay uninterruptedly with their partners and away from those people who can give help or at least validate their experiences and, particularly if these women live in small houses (Bradbury-Jones and Isham, 2020; Mazza M. et al., 2020; van Gelder et al., 2020). Indeed, some studies suggest that sudden forced proximity with their immediate household members is a risk factor for domestic violence, and aggression (Taylor et al., 2008; Brooks et al., 2020). In Italy, since the beginning of the COVID-19 quarantine, three domestic homicides and 11 murder-suicides have been registered to date.

Furthermore, while COVID-19 has coincided with greater rises in unemployment for women than men, the rise in unemployment for men remains substantial (Bureau of Labor Statistics, 2020).

About the age groups, depressive symptoms were most likely to occur in younger people (aged 18–29 years). With increasing age, depressive symptoms were less prevalent during the Italian lockdown due to the COVID-19 outbreak. Our results were similar to those from previous studies, such as a study during the SARS outbreak in Taiwan (Su et al., 2007), a study of horse owners quarantined because of equine influenza (Taylor et al., 2008), and one recent study during the COVID-19 epidemic in China (Huang and Zhao, 2020). As well, like gender, for this sociodemographic variable, the prevalence of the depressive symptoms in different age groups and the probability of risk of developing depressive symptoms depending on age do not relate to the greater likelihood of being infected.

Being elderly has been reported to correlate with adverse clinical outcomes, including hospitalization and mortality (Applegate and Ouslander, 2020; Zhou et al., 2020). Indeed, in Italy the mean age of COVID-19 patients who died was 81 years (Remuzzi and Remuzzi, 2020) and the case fatality rate was 16% from 60 to 79 years, 19.7% from 80 to 89 years, and 16% for 90 years and older (Livingston and Bucher, 2020). Despite this, respondents older than 60 years had the lowest risk for developing depressive symptoms compared to the younger age groups. In a population where loneliness and isolation have already been described as an epidemic (Luo et al., 2012), the impact of even short-term social distancing measures and the resulting distress did not influence the vulnerability to mental health issues (Jeste et al., 2020; Vahia et al., 2020). This finding is in accordance with part of the literature. Although mixed results derive across current and previous studies on the association between participants’ age and depression as a psychological outcome of health-related emergency (Hawryluck et al., 2004; Qiu et al., 2020), some authors reported that only young age was found to be associated with increased distress as a psychological outcome of the COVID-19 quarantine (Barari et al., 2020; Mazza C. et al., 2020) and of the SARS quarantine (Hawryluck et al., 2004). The higher psychological distress reported by the younger population could be due to their greater and uncontrolled access to the amount of information (“*infodemic*”) through social media, which can easily trigger distress (Cheng et al., 2014).

As regards marital status, unmarried/single people were the most depressed group with quarantine policies in the event of this outbreak. It is plausible that single people had greater difficulty in relying on or obtaining the assistance of others during the Italian lockdown, thus are at risk of depressive symptoms and lower self-confidence more than cohabiting and married participants. This datum is in line with part of previous literature reporting that being married was protective for depression or associated with a lower risk of depressive symptoms (Inaba et al., 2005; Yan et al., 2011; Bulloch et al., 2017), although other studies conducted during the SARS outbreak suggested that demographic factors such as marital status, as well as living with other adults, and having children were not associated with psychological outcomes (Hawryluck et al., 2004; Mihashi et al., 2009).

As to geographic area of residence, people living in the south of Italy showed the lowest risk of developing depressive symptoms among all the groups, followed by participants from regions of central Italy compared to residents from northern regions and the islands. As expected, residents in the most severely affected regions are at the highest risk of developing depressive symptoms. Southern and central regions recorded a smaller number of deaths and diagnosed cases (1,812 and 2,730 deaths, respectively), compared to the north-east and north-west regions (6,935 and 21,009 deaths, respectively), where the disease spread first on a large scale. To explain this datum, it should be considered that the authorities introduced control measures in the northern regions (the “Red Zone”), before any other region and carried out extraordinary efforts to restrict the movement of people (Carlucci et al., 2020). In addition, residents from northern Italy were found less adherent to restrictive measures compared to the those from the south of Italy. People who have shown more adherence were found less at risk of depressive symptoms compared to people with less adherence (see under).

As for occupational status, this study highlighted students as suffering from the highest level of psychological distress among all the other groups, including the unemployed group. Also, in this case, the public’s level of depressive symptoms did not increase with an increased probability of contracting the disease. Since the physical spaces of universities were closed, students’ mental well-being was affected by the sudden interruption of social interactions. However, the possibility of having online lessons and maintaining social contacts through social networks would not explain the onset of the depressive symptoms compared to other groups, for example employees who had been laid off or were retired.

The reason for students’ greater risk of depressive symptoms, reported also by Wang et al. (2020) among the Chinese population, could lie in a sense of uncertainty toward the future that this emergency, not only in health, but also in economic, social, and political areas, is eliciting all over the world (Chong et al., 2004; Wenzel et al., 2005; Tan and Enderwick, 2006).

Compared with other professions and the general population, healthcare workers were associated with a lower risk of psychological outcomes compared to the unemployed in our sample. “Learned helplessness”(Seligman, 1972) could explain why health professionals were the least depressed group. After being exposed to inescapable difficult events, people become passive and stop trying after being exposed to events such as uncontrollable bursts of noise (Alloy et al., 1984) and as a result show greater levels of anxiety and depression. On the contrary, health professionals, considered the real heroes of this emergency, were associated with a lower risk of psychological outcomes compared to unemployed participants. Through the practice of their profession, they felt more useful to society, despite their increased risk for infection and transmission (Al-Rabiaah et al., 2020).

After students, the unemployed were at a higher risk of depressive symptoms compared to the other groups (Stuckler et al., 2009; Reeves et al., 2012). This datum is inserted within the context of the COVID-19-related risk unemployment and economic losses and insecurity with the closure of community services and the collapse of industries negatively impacting the national economy. It should be a critical public health priority to prevent suicide. Indeed, during the most recent economic recession, a 1% rise in unemployment was correlated with a rise in the suicide rate of 0.99% in the United States (95% CI: 0.60–1.38, *p* < 0.001) (Reeves et al., 2012). Similarly, each percentage point increase in unemployment was accompanied by 0.79% rise in suicide (95% CI: 0.16–1.42, p = 0.016) in Europeans aged 65 years or less.

As to adherence, people with low adherence were more likely to exhibited depressive symptoms relative to people with a great level of adherence to COVID-19 preventive measures. As expected, adherence has been found to be a protective factor against mental health problems (Hawryluck et al., 2004; Koenig and Schultz, 2010; Brooks et al., 2020). Adopting the preventive behaviors contribute to lower the uncertainty of the epidemic progression which would cause higher psychological pressure on the public. As to worry about being infected with COVID-19, people with more worry were more depressed than people with a low level of worry.

As for the whole sample, also in the subsample of participants with psychotherapeutic treatment history, the same sociodemographic factors and behaviors that contribute to, or mitigate mental effects of the quarantine in terms of depressive symptoms were reported. However, when anxiety symptoms or concomitant stressful events were present in comorbidity, these participants were found to experience higher levels of worry associated with increased depression symptomatology, compared to the whole sample. Current evidence showed similar results. A history of stressful situations and medical problems was associated with a greater degree of depression and anxiety during the COVID-19 quarantine in the Italian population (Hao et al., 2020). Again, psychiatric patients were significantly more likely to experience a higher degree of the negative mental impact of the outbreak, including stress, anxiety, and depression, compared to the general public (Hao et al., 2020).

## Conclusion

During the COVID-19 quarantine outbreak in Italy, female participants, younger people, single people, students, people living in northern regions, and who were less compliant with quarantine guidelines and less worried about being infected with COVID-19 were at a high risk of displaying psychological issues. These findings suggest public levels of depressive symptoms did not increase with the greater likelihood of being infected. For example, although female and younger people reported a lower risk of COVID-19 infection, they experienced higher levels of depressive symptoms during the COVID-19 quarantine in Italy.

Therefore, ongoing monitoring of the psychological strain associated with outbreaks of epidemic-potential, life-threatening diseases should become routine as part of preparedness efforts worldwide by establishing early targeted mental health interventions. In other words, more vulnerable groups, likes those cited above, should benefit from personalized “morale-boosting” interventions. Or, intervention research could be valuable to combat amplifications of gender inequalities, particularly to address the added challenges women are likely to face. This research can provide timely insights for government agencies toward improving and safeguarding the psychological well-being of women, younger people, and categories of subjects at a higher risk of suffering from psychological distress on the occasion of subsequent waves of the spread of COVID-19 or other epidemic diseases. This study has several limitations. Firstly, the analyses presented here were derived from a cross-sectional design, thus it is difficult to make causal inferences. Secondly, given that the research was conducted in close temporal proximity to the period of the COVID-19 quarantine, a web-based survey method was necessary to recruit a convenience sample by avoiding possible infections. This limited sampling in our study. As a consequence, a self-selection effect may have occurred and should be considered with those people who were experiencing the greatest or least levels of distress responding to the survey (Saggino et al., 2017). In addition, participants were required to access the internet and to be familiar with online devices to respond, which suggests that they might be more educated, younger or/and have a higher socioeconomic status than the overall surveyed quarantined population. Thirdly, due to the uncontrolled occurrence of this health-related emergency, an accurate picture of the individual’s psychological conditions before the COVID-19 outbreak was not conducted. Although it would been interesting to conduct pre-post analyses, these data could provide a baseline for future research on the psychological consequences of quarantine in the Italian population throughout the rest of the current COVID-19 pandemic. Fourthly, depressive and state anxiety symptoms were measured by means self-report inventories that are notably biased by response set, such as social desirability (Innamorati et al., 2014; Carlucci et al., 2015; Balsamo et al., 2018a,b). Thus, future research should include methods, such as observational methods and psychophysiological or behavioral assessment, in order to objectively record the levels of these mood states (Campbell and Fiske, 1959). Fifthly, the assessment of state anxiety could be completed or replaced by adding the specific fear of COVID-19 scale (Ahorsu et al., 2020; Broche-Pérez et al., 2020; Soraci et al., 2020). In addition, it should be acknowledged that the study was carried out was not sufficiently heterogeneous for sex, with marked female preponderance, and age sample, with a prevalence of juveniles. Hence, these findings may not translate accurately to the public at large.

Finally, it could not evaluate whether the outcomes considered in this study will be long-lasting after the COVID-19 outbreak. However, follow-up with these participants will continue in order to facilitate our understanding about how long these outcomes will last. A deeper understanding of how the epidemic affects Italians’ psychological health by identifying which groups were at a high risk of psychological morbidities due to the current pandemic can help to guide and target future psychological intervention implementations.

## Data Availability Statement

The raw data supporting the conclusions of this article will be made available by the authors, without undue reservation, to any qualified researcher.

## Ethics Statement

The studies involving human participants were reviewed and approved by the Department of Psychological Sciences, Health and Territory, University of Chieti, Italy, Review Board. The patients/participants provided their written informed consent to participate in this study.

## Author Contributions

Both authors listed have made a substantial, direct and intellectual contribution to the work, and approved it for publication.

## Conflict of Interest

The authors declare that the research was conducted in the absence of any commercial or financial relationships that could be construed as a potential conflict of interest.
