# Supplementary material for: Italians on the Age of COVID-19: The Self-Reported Depressive Symptoms Through Web-Based Survey
Source: Front Psychol. 2020 Oct 16;11:569276. doi: 10.3389/fpsyg.2020.569276 (PMC7596268; doi:10.3389/fpsyg.2020.569276)
Supplement: Supplementary file 1 [file Data_Sheet_1.docx]

Appendix A. Results of GLMs model 2 controlled for anxiety (N=3,672).

| **Characteristic** |  | **Depressive symptoms*Anxiety** | | | | | | |
| --- | --- | --- | --- | --- | --- | --- | --- | --- |
|  |  | **β** | **SE** | **Wald χ^2^** | **Sign.** | **Exp(β)** | **95% CI Exp(β)** | |
| *Sex* |  |  |  |  |  |  |  |  |
| Female |  | .207 | .073 | 7.991 | .005**** | 1.230 | 1.066 | 1.42 |
| Male |  |  |  |  |  | 1 [Reference] |  |  |
|  |  |  |  |  |  |  |  |  |
| *Age* |  |  |  |  |  |  |  |  |
| over 60 |  | -.478 | .199 | 5.73 | *.017*** | .620 | .419 | 0.917 |
| 50-59 |  | -.393 | .167 | 5.532 | *.019*** | .675 | .487 | 0.937 |
| 40-49 |  | -.271 | .132 | 4.186 | *.041*** | .763 | .589 | 0.989 |
| 30-39 |  | -.293 | .103 | 8.031 | *.005*** | .746 | .610 | 0.914 |
| 18-29 |  |  |  |  |  | 1 [Reference] |  |  |
|  |  |  |  |  |  |  |  |  |
| *Marital Status* |  |  |  |  |  |  |  |  |
| Widowed |  | -.762 | .3148 | 5.864 | *.015*** | .467 | .252 | 0.865 |
| Cohabiting |  | -.217 | .1148 | 3.57 | .059 | .805 | .643 | 1.008 |
| Divorced/separated |  | -.425 | .2084 | 4.164 | *.041*** | .654 | .434 | .983 |
| Married |  | -.364 | .1126 | 10.42 | *.001** | .695 | .557 | .867 |
| Single |  |  |  |  |  | 1 [Reference] |  |  |
|  |  |  |  |  |  |  |  |  |
| *Geographic Area* |  |  |  |  |  |  |  |  |
| Islands |  | .198 | .154 | 1.64 | .200 | 1.219 | .900 | 1.651 |
| South |  | -.190 | .083 | 5.151 | *.023*** | .827 | .702 | .974 |
| Central |  | -.230 | .109 | 4.462 | *.035*** | .794 | .641 | .984 |
| Nord-East |  | .122 | .127 | .923 | .337 | 1.13 | .881 | 1.45 |
| Nord-West |  |  |  |  |  | 1 [Reference] |  |  |
|  |  |  |  |  |  |  |  |  |
| *Occupational Status* |  |  |  |  |  |  |  |  |
| Employee |  | -.107 | .110 | .944 | .331 | .899 | .724 | 1.115 |
| Healthcare professional |  | -.151 | .166 | .825 | .364 | .860 | .621 | 1.191 |
| Student |  | .256 | .124 | 4.259 | *.039*** | 1.292 | 1.013 | 1.647 |
| Retired |  | -.060 | .250 | .057 | .812 | .942 | .576 | 1.540 |
| Unemployed |  |  |  |  |  | 1 [Reference] |  |  |
|  |  |  |  |  |  |  |  |  |
| *Adherence to Quarantine* |  |  |  |  |  |  |  |  |
| High |  | -.397 | .070 | 31.789 | *.001** | .673 | .586 | .772 |
| Low |  |  |  |  |  | 1 [Reference] |  |  |
|  |  |  |  |  |  |  |  |  |
| *COVID-19- related worry* |  |  |  |  |  |  |  |  |
| None |  | .023 | .134 | .029 | .866 | .978 | .752 | 1.271 |
| Moderate |  | .224 | .138 | 2.613 | *.001** | 1.252 | .953 | 1.643 |
| Quite a lot |  |  |  |  |  | 1 [Reference] |  |  |

** p<.001; ** p<.05*

*Note.*

The Exp(β) or Odds ratio and β values (95%Wald CI) were derived from generalized linear regression (logistic ordinal)

COVID-19 = coronavirus disease 2019.

Appendix B. Results of GLMs model 1 and 2 for the subsample of participants with psychotherapeutic treatment history.

| **Characteristic** |  | **Depressive symptoms** | | | | | | |  | **Depressive symptoms*Anxiety** | | | | | | |
| --- | --- | --- | --- | --- | --- | --- | --- | --- | --- | --- | --- | --- | --- | --- | --- | --- |
|  |  | **β** | **SE** | **Wald χ^2^** | **Sign.** | **Exp(β)** | **95% CI Exp(β)** | |  | **β** | **SE** | **Wald χ^2^** | **Sign.** | **Exp(β)** | **95% CI Exp(β)** | |
| *Sex* |  |  |  |  |  |  |  |  |  | | | | | | | |
| Female |  | .310 | .151 | 4.220 | *.040*** | 1.364 | 1.014 | 1.834 |  | -.144 | .155 | .864 | .353 | .866 | .639 | 1.173 |
| Male |  |  |  |  |  | 1 [Reference] |  |  |  |  |  |  |  | 1 [Reference] |  |  |
|  |  |  |  |  |  |  |  |  |  |  |  |  |  |  |  |  |
| *Age* |  |  |  |  |  |  |  |  |  |  |  |  |  |  |  |  |
| over 60 |  | -.521 | .359 | 2.107 | .147 | .594 | .294 | 1.200 |  | -.050 | .430 | .014 | .907 | .951 | .409 | 2.210 |
| 50-59 |  | -.696 | .339 | 4.192 | *.041*** | .499 | .256 | .971 |  | -.492 | .344 | 2.038 | .153 | .611 | .311 | 1.201 |
| 40-49 |  | -.430 | .235 | 3.332 | .068 | .651 | .410 | 1.032 |  | -.177 | .239 | .546 | .460 | .838 | .524 | 1.339 |
| 30-39 |  | -.543 | .187 | 8.406 | *.004*** | .581 | .403 | .839 |  | -.504 | .194 | 6.735 | *.009*** | .604 | .413 | 0.884 |
| 18-29 |  |  |  |  |  | 1 [Reference] |  |  |  |  |  |  |  | 1 [Reference] |  |  |
|  |  |  |  |  |  |  |  |  |  |  |  |  |  |  |  |  |
| *Marital Status* |  |  |  |  |  |  |  |  |  |  |  |  |  |  |  |  |
| Widowed |  | -1.682 | .911 | 3.402 | .065 | .186 | .031 | 1.111 |  | -1.464 | 1.155 | 1.605 | .205 | .231 | .024 | 2.227 |
| Cohabiting |  | -.526 | .193 | 7.416 | *.006*** | .591 | .404 | .863 |  | -.303 | .202 | 2.245 | .134 | .738 | .496 | 1.098 |
| Divorced/separated |  | -.362 | .386 | .877 | .349 | .696 | .327 | 1.485 |  | -.398 | .354 | 1.263 | .261 | .672 | .335 | 1.345 |
| Married |  | -.446 | .199 | 5.004 | *.025*** | .640 | .433 | .946 |  | -.249 | .213 | 1.357 | .244 | .780 | .513 | 1.185 |
| Single |  |  |  |  |  | 1 [Reference] |  |  |  |  |  |  |  | 1 [Reference] |  |  |
|  |  |  |  |  |  |  |  |  |  |  |  |  |  |  |  |  |
| *Geographic Area* |  |  |  |  |  |  |  |  |  |  |  |  |  |  |  |  |
| Islands |  | .304 | .322 | .886 | .346 | 1.355 | .720 | 2.549 |  | .316 | .303 | 1.081 | .298 | 1.371 | .756 | 2.486 |
| South |  | -.224 | .161 | 1.909 | .167 | .800 | .582 | 1.098 |  | -.373 | .170 | 4.778 | *.029*** | .688 | .493 | 0.962 |
| Central |  | -.045 | .188 | .057 | .811 | .956 | .661 | 1.382 |  | -.192 | .200 | .916 | .338 | .826 | .558 | 1.222 |
| Nord-East |  | .134 | .198 | .456 | .499 | 1.143 | .775 | 1.687 |  | .045 | .213 | .044 | .834 | 1.046 | .688 | 1.590 |
| Nord-West |  |  |  |  |  | 1 [Reference] |  |  |  |  |  |  |  | 1 [Reference] |  |  |
|  |  |  |  |  |  |  |  |  |  |  |  |  |  |  |  |  |
| *Occupational Status* |  |  |  |  |  |  |  |  |  |  |  |  |  |  |  |  |
| Employee |  | -.069 | .212 | .104 | .747 | .934 | .615 | 1.417 |  | -.073 | .215 | .116 | .733 | .929 | .609 | 1.417 |
| Healthcare professional |  | -.797 | .280 | 8.095 | *.004*** | .450 | .260 | .780 |  | -.536 | .282 | 3.593 | .058 | .585 | .336 | 1.018 |
| Student |  | .067 | .234 | .081 | .776 | 1.069 | .675 | 1.692 |  | -.009 | .240 | .001 | .970 | .991 | .618 | 1.588 |
| Retired |  | -.455 | .539 | .714 | .398 | .634 | .221 | 1.824 |  | -1.018 | .613 | 2.753 | .097 | .361 | .108 | 1.203 |
| Unemployed |  |  |  |  |  | 1 [Reference] |  |  |  |  |  |  |  | 1 [Reference] |  |  |
|  |  |  |  |  |  |  |  |  |  |  |  |  |  |  |  |  |
| *Adherence to Quarantine* | |  |  |  |  |  |  |  |  |  |  |  |  |  |  |  |
| High |  | -.475 | .133 | 12.574 | *.001** | .622 | .479 | .809 |  | -.237 | .141 | 2.779 | .096 | .789 | .598 | 1.042 |
| Low |  |  |  |  |  | 1 [Reference] |  |  |  |  |  |  |  | 1 [Reference] |  |  |
|  |  |  |  |  |  |  |  |  |  |  |  |  |  |  |  |  |
| *COVID-19- related worry* | |  |  |  |  |  |  |  |  |  |  |  |  |  |  |  |
| None |  | -.303 | .2375 | 1.628 | .202 | .739 | .464 | 1.176 |  | .544 | .246 | 4.870 | *.027*** | 1.722 | 1.063 | 2.791 |
| Moderate |  | -.010 | .1425 | .005 | .941 | .990 | .749 | 1.308 |  | .564 | .157 | 12.873 | *.001** | 1.757 | 1.292 | 2.391 |
| Quite a lot |  |  |  |  |  | 1 [Reference] |  |  |  |  |  |  |  | 1 [Reference] |  |  |

** p<.001; ** p<.05*

*Note.*

The Exp(β) or Odds ratio and β values (95%Wald CI) were derived from generalized linear regression (logistic ordinal)

COVID-19 = coronavirus disease 2019.
